# Context-dependent immunoregulation by BAFF: reconciling its pro-tumorigenic, anti-tumor, and autoimmune roles through a multi-factorial lens

**DOI:** 10.3389/fimmu.2026.1975879

**Published:** 2026-10-28

**Authors:** Jiayi Shang, Minli Jin, Kui Jiang, Weiqi Liu, Jinji Li, Xiaochi Chen

**Affiliations:** 1 The First Affiliated Hospital of Dalian Medical University, Dalian, China; 2 Dalian Medical University, Dalian, China; 3 Department of Urology, First Hospital of Shanxi Medical University, Taiyuan, China; 4 Department of Gastroenterology, Shengjing Hospital of China Medical University, Shenyang, China; 5 Department of Cardiology, Dandong First Hospital, Dandong, China

**Keywords:** autoimmune diseases, BAFF, BAFF receptors, B-cell lymphoma, biomarker, precision medicine, targeted therapy, tumor microenvironment

## Abstract

B cell activating factor (BAFF), originally characterized as a canonical survival factor for B cells, is now recognized as a pleiotropic immunoregulator that bridges innate and adaptive immunity. Its biological effects are highly context-dependent, manifesting as promotion of malignant B-cell survival in hematological malignancies, conflicting activities in solid tumors, and disruption of B-cell tolerance in autoimmune diseases. Current literature has largely examined BAFF within disease-specific frameworks, leaving cross-disease mechanistic relationships inadequately resolved. In this review, we propose a multi-factorial framework for integrating evidence across cancer and autoimmunity through four proposed variables: ligand concentration, oligomeric form, cellular source, and receptor availability. By dissecting receptor signaling via BAFF receptor (BAFF-R), transmembrane activator and calcium modulator and cyclophilin ligand interactor (TACI), and B cell maturation antigen (BCMA), we evaluate the therapeutic landscape, safety profiles, and combination strategies of BAFF-targeted agents. The framework is a working hypothesis, not a validated causal model. We propose three integrated perspectives: (i) a proposed multi-factorial framework for interpreting BAFF’s divergent functions; (ii) a conceptual, testable inverted U-shaped working hypothesis for solid tumors, without validated concentration thresholds; and (iii) the translational utility of BAFF system molecules as stratification biomarkers and precision therapeutic targets. Autoimmune disease data provide conceptual support for this concentration-form-context framework, which was initially developed to reconcile contradictory observations in tumors. The framework requires direct experimental titration and oligomer-specific validation. Finally, we identify critical knowledge gaps, including distinct signaling pathways elicited by BAFF oligomers and crosstalk with inflammatory networks, to inform next-generation personalized immunotherapy.

## 1 Introduction

BAFF is a member of the tumor necrosis factor superfamily. Since its discovery, it has been recognized as an essential survival factor for B cells, regulating their proliferation and maturation (1). Over the past decade, however, accumulating evidence has revealed that the functions of BAFF extend well beyond this classical role. BAFF is now understood as a pleiotropic immunoregulatory molecule whose dysregulation underpins a range of pathological conditions. It promotes follicular helper T-cell (Tfh) differentiation, modulates innate immunity through effects on neutrophils and dendritic cells, and remodels the tumor microenvironment in both hematological malignancies and solid tumors (1). Elevated BAFF levels correlate with disease activity and treatment outcomes in various B-cell lymphomas. The approval of belimumab, a BAFF-targeting antibody, for systemic lupus erythematosus (SLE) has further established the clinical relevance of this pathway (1).

In autoimmune diseases such as SLE, Sjögren’s syndrome (SS), and rheumatoid arthritis (RA), elevated BAFF levels represent a hallmark of pathology (2, 3). Yet this functional breadth raises a fundamental question. How can a single ligand-receptor system coordinate such diametrically opposed immune outcomes? Recently, Li et al. provided a comprehensive overview of the BAFF system in autoimmune diseases (4). That review effectively synthesizes mechanisms within the autoimmune domain. However, the broader literature reveals a persistent silo effect, with BAFF studied intensively within individual disease categories including autoimmunity, hematological malignancy, and solid tumors.

Systematic cross-disease integration remains scarce, and a critical gap therefore persists. BAFF signals through the same ligand–receptor system in both malignant and autoimmune microenvironments, yet produces starkly divergent outcomes. The same cytokine supports malignant B-cell survival in lymphoma while breaking B-cell tolerance in lupus. This cross-disease paradox cannot be resolved by disease-specific reviews alone. Moreover, the paradoxical pro- versus anti-tumor effects of BAFF in solid tumors remain insufficiently clarified, and this topic is largely absent from autoimmune-focused reviews.

Equally underexplored is the potential of BAFF system molecules to serve as clinical biomarkers across the cancer–autoimmunity spectrum. To address these gaps, this review integrates BAFF’s dual pathological roles across cancer and autoimmunity. We apply a proposed concentration-form-context framework to evaluate therapeutics and biomarker potential.

This narrative review was conducted according to a structured, non-systematic search strategy. PubMed/MEDLINE, Web of Science and Embase were searched from database inception to August 2026 using combinations of the terms “BAFF”, “BLyS”, “TNFSF13B”, “BAFF-R/TNFRSF13C”, “TACI/TNFRSF13B”, “BCMA/TNFRSF17”, together with disease-specific and mechanism-specific keywords (e.g., “B-cell lymphoma”, “solid tumor”, “tertiary lymphoid structure”, “systemic lupus erythematosus”, “Sjogren”, “neuromyelitis optica”, “CAR-T”, “biomarker”). Reference lists of retrieved articles and relevant reviews were screened manually for additional primary studies. Seminal pre-2020 primary studies, including the characterization of BAFF-transgenic mice (5) and the identification of TACI and BCMA as BAFF/APRIL receptors (6), were included to anchor the mechanistic framework. No language restriction other than English was applied. Given the hypothesis-driven scope of this review, this was a targeted literature search rather than a systematic review, and the evidence level of each cited study is classified in the “Clarification of evidence levels” paragraph below. Regulatory and developmental-status statements, including trial phase, approval dates, and BLA or PDUFA timelines, are sourced from ClinicalTrials.gov, FDA, NMPA, or EMA public databases, or from product labels as cited. Non-peer-reviewed company announcements were not used as evidence of efficacy or mechanism. Where no peer-reviewed or official regulatory source was available, the statement was deleted or restricted to a development-status note.

Beyond the disease-by-disease synthesis of Li et al. (4), the present review contributes three elements: (i) a mechanism-first, four-variable framework proposed to help explain BAFF’s divergent outputs across oncology and autoimmunity within a single schema; (ii) a testable, falsifiable concentration-form-context prediction (the inverted-U window) that is absent from autoimmune-focused reviews; and (iii) a proposed therapeutic-selection logic that links local versus systemic BAFF exposure and oligomeric state to the choice between ligand blockade, receptor blockade, and agonistic exploitation of BAFF biology.

Clarification of evidence levels. Throughout this review, we classify clinical and mechanistic statements into four categories. Demonstrated mechanisms are supported by direct experimental manipulation in the cited primary literature, such as genetic knockout, receptor titration, or oligomer-specific perturbation. Proposed or inferred mechanisms are derived from correlative observations, computational models, or cross-disease analogy and are explicitly identified as such. Correlative clinical associations describe relationships observed in patient cohorts without establishing causality. Regulatory or development status statements are reported only as status and are not used as evidence of clinical efficacy. Specifically, disease-specific oligomeric assignments in **Table 1**, the receptor-associated NF-kB signaling scheme in **Figure 1**, and the inverted U-shaped concentration-effect hypothesis in **Figure 2** are classified as proposed or inferred rather than directly validated in native diseased human tissues.

**Table 1:** Cross-disease mechanistic mapping of BAFF functions.

Mechanistic axis	Hematological malignancies	Solid tumors (pro-tumor)	Solid tumors (anti-tumor)	SLE	pSS	NMOSD
Ligand concentration	Relative high (systemic and local; conceptual) (53)	Relative high (TME/CAF-derived; conceptual) (56)	Relative moderate (transient local; conceptual) (64)	Relative high (systemic; conceptual) (46)	Relative high (local glandular; conceptual) (69)	Relative high (BM neutrophil-derived; conceptual) (70)
Proposed or inferred oligomeric context	Inferred:60-mer or membrane-bound	Inferred:60-mer or membrane-bound	Inferred: soluble trimer	Inferred: soluble trimer or 60-mer	Inferred: soluble trimer or 60-mer	Inferred: soluble trimer
Cellular source	Neutrophil-stromal axis (53)	CAFs, tumor cells, MDSCs (41, 55, 56)	Tumor-associated neutrophils (67)	Neutrophils, cDCs, monocytes (12, 46)	Salivary epithelial cells, pDCs (69)	ISG15+ neutrophils (70)
Target cell fate	Malignant B cell survival (47–50)	Breg expansion, T-cell suppression (41, 55)	TIB survival, TLS formation, ADCC (60, 64)	Autoreactive B cell rescue, Tfh expansion (1, 37)	Ectopic GC formation, local autoantibody (69)	ASC maturation, autoantibody (70)
Net outcome	Pro-tumor (47–50)	Pro-tumor (55–57)	Anti-tumor (context-dependent) (64)	Pathogenic (46)	Pathogenic (local) (69)	Pathogenic (70)
Therapeutic implication	BAFF-R blockade and BAFF-directed CAR-T/TCE approaches (1, 68)	Blockade?/Caution	Potentially support?	Blockade (belimumab, telitacicept) (87, 95)	Blockade (104)	Blockade

The same four proposed variables may jointly influence BAFF’s functional output across oncological and autoimmune settings, supporting a conceptual, mechanism-based classification.

BAFF, B cell activating factor; TME, tumor microenvironment; CAF, cancer-associated fibroblast; MDSC, myeloid-derived suppressor cell; TIB, tumor-infiltrating B cell; TLS, tertiary lymphoid structure; ADCC, antibody-dependent cellular cytotoxicity; SLE, systemic lupus erythematosus; pSS, primary Sjögren’s syndrome; NMOSD, neuromyelitis optica spectrum disorder; GC, germinal center; ASC, antibody-secreting cell; BM, bone marrow; pDC, plasmacytoid dendritic cell; cDC, conventional dendritic cell.

Note on oligomeric assignments: Disease-specific oligomeric assignments are proposed or inferred from *in vitro* binding studies and computational models rather than direct biochemical quantification in native diseased tissues. Oligomer-specific assays in clinical samples are needed to validate these assignments.

**Figure 1:**
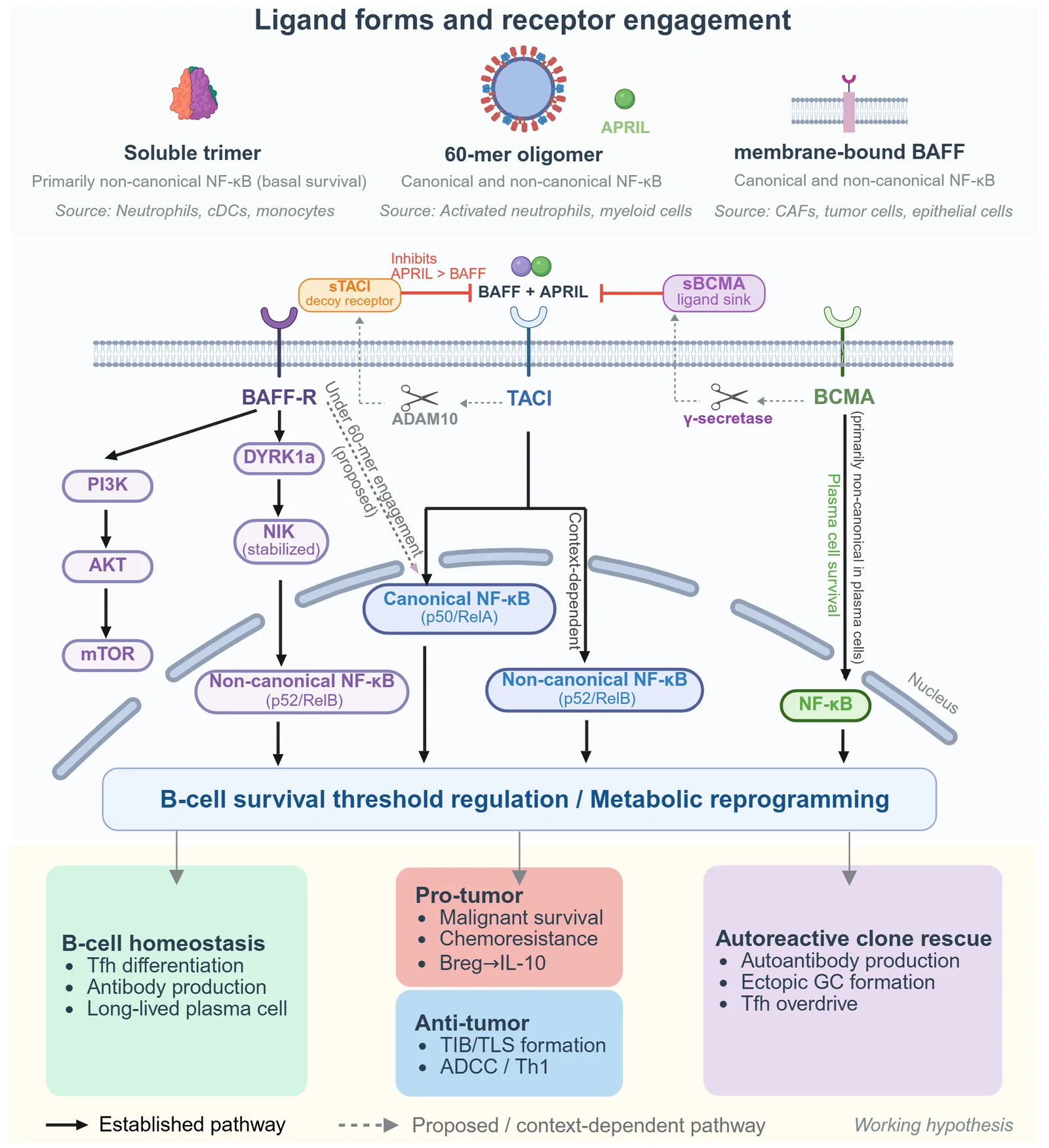
This schematic presents a working-hypothesis model integrating established and proposed BAFF signaling mechanisms. BAFF exists in three oligomeric states—soluble trimers (derived primarily from neutrophils, cDCs, and monocytes), 60-mer oligomers (from activated neutrophils and myeloid cells), and membrane-bound BAFF (from CAFs, tumor cells, and epithelial cells)—that differentially engage the three receptors BAFF-R, TACI, and BCMA. Soluble trimers primarily signal through BAFF-R via the non-canonical NF-κB pathway (DYRK1A-mediated NIK stabilization) and the PI3K/AKT/mTOR axis to maintain basal B-cell survival. TACI activates both canonical (p50/RelA) and non-canonical (p52/RelB) NF-κB in a context-dependent manner. BCMA drives NF-κB signaling to maintain plasma cell survival. Notably, under 60-mer engagement, BAFF-R may also activate canonical NF-κB signaling; this pathway is proposed based on *in vitro* and in silico evidence (31) and requires validation in native tissues. Proteolytic shedding generates soluble decoy receptors that negatively regulate ligand availability: ADAM10 cleaves TACI to release sTACI (preferentially inhibiting APRIL over BAFF), whereas γ-secretase cleaves BCMA to release sBCMA (acting as a ligand sink). The net signaling output is proposed to influence B-cell survival thresholds and metabolic reprogramming, directing context-dependent functional consequences ranging from physiological B-cell homeostasis and humoral immunity to pro-tumor immune escape, anti-tumor tertiary lymphoid structure (TLS) formation and ADCC, and autoimmune tolerance breach. Arrow styles denote evidence levels: solid arrows indicate experimentally established pathways; dashed arrows indicate proposed or context-dependent interactions. Created in BioRender. Shang, J. (2026) https://BioRender.com/o41nf8e.

**Figure 2:**
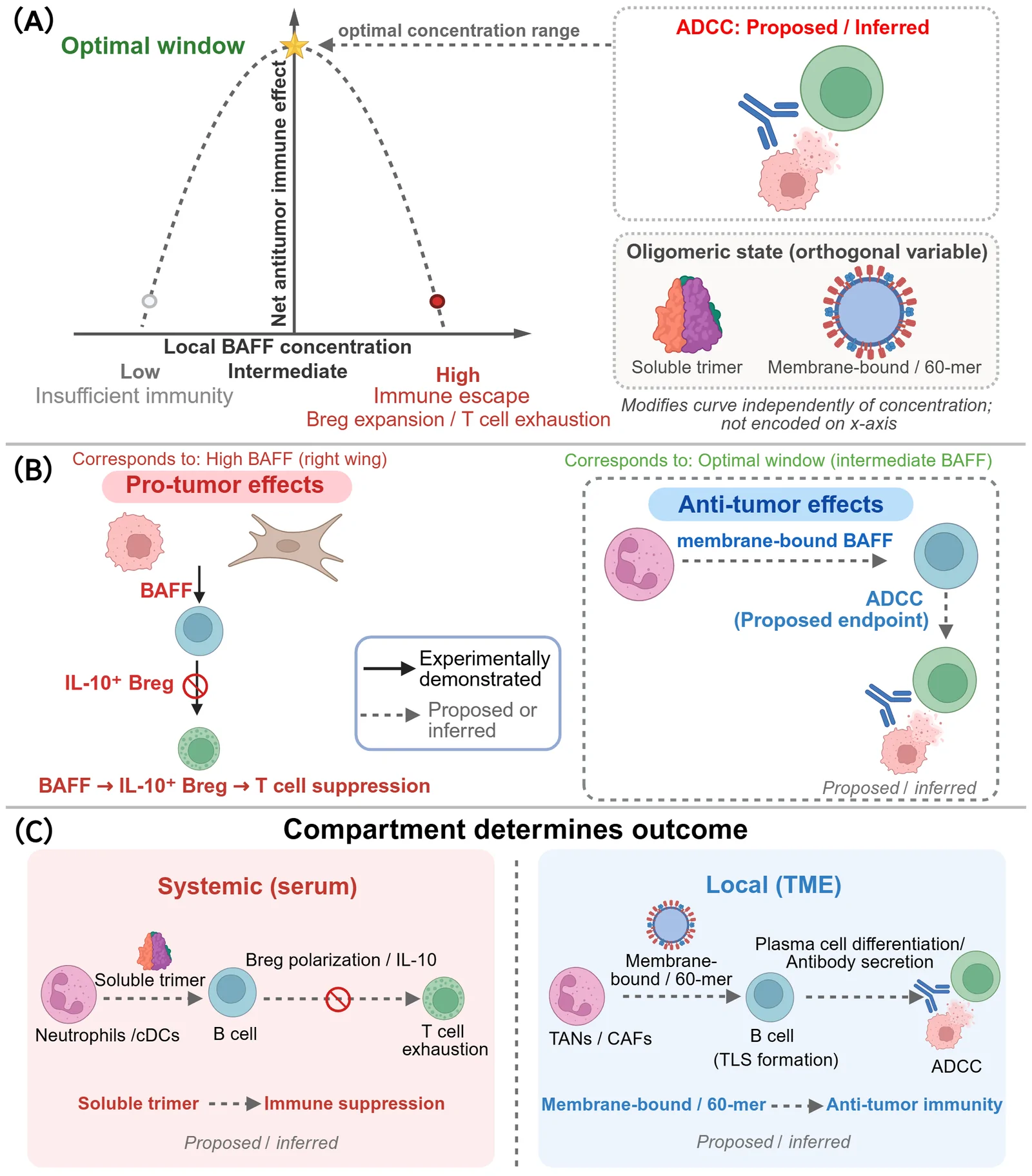
**(A)** Concentration–effect curve. The net antitumor immune effect of BAFF is proposed to follow an inverted U-shaped function of local BAFF concentration. At low concentrations, local humoral immunity is insufficient. An intermediate “optimal window” supports antitumor immunity through TIB survival, TLS formation, and Th1 polarization. At high concentrations, chronically elevated BAFF promotes immune escape via Breg expansion and T-cell exhaustion. The x-axis concentration zones (Low/Intermediate/High) represent conceptual categories and do not correspond to validated concentration thresholds. The pro-tumor arm illustrated in **(B)** (left)—tumor- and CAF-derived BAFF driving IL-10^+^ Breg expansion and T-cell suppression in cervical cancer—is experimentally demonstrated (41). The anti-tumor arm [**(B)**, right]—membrane-bound BAFF from tumor-associated neutrophils driving plasma cell differentiation and ADCC—is proposed or inferred (67); the optimal window, the curve shape, and the positioning of ADCC at the intermediate concentration range are hypothetical and require direct *in vivo* validation. The ADCC endpoint is explicitly labeled as proposed/inferred. Oligomeric composition (membrane-bound/60-mer-enriched versus soluble-trimer-rich) is an orthogonal modifier of this curve and is not encoded on the concentration axis. **(B)** Mechanistic schematics corresponding to concentration intervals. The pro-tumor arm (left) corresponds to the high-BAFF right wing of the curve: tumor cells and CAFs secrete BAFF, driving IL-10^+^ Breg expansion and T-cell suppression, as observed in cervical cancer models. The anti-tumor arm (right) corresponds to the optimal window (intermediate BAFF): membrane-bound BAFF from tumor-associated neutrophils is proposed to drive plasma cell differentiation and ADCC. Solid arrows indicate experimentally demonstrated pathways; dashed arrows indicate proposed or inferred mechanisms. **(C)** Local versus systemic BAFF exposure. The same ligand produces divergent outcomes depending on compartment. Systemic, neutrophil/cDC-derived soluble BAFF (left) is proposed to favor Breg polarization, IL-10 production, and T-cell exhaustion, leading to immune suppression. Locally membrane-bound/60-mer-enriched BAFF within the TME (right) is proposed to support TLS formation, plasma cell differentiation, antibody secretion, and ADCC, promoting anti-tumor immunity, and is likewise drawn with dashed arrows. As in panel B, solid arrows denote experimentally demonstrated pathways and dashed arrows denote proposed or inferred mechanisms. Created in BioRender. Shang, J. (2026) https://BioRender.com/s69626m.

## 2 Molecular biology and signaling network of BAFF

### 2.1 Gene, protein structure, and expression regulation of BAFF

BAFF is also known as tumor necrosis factor superfamily member 13B (TNFSF13B). It is a key member of the TNF superfamily and plays a central role in B-cell survival, development, and immunoglobulin secretion (7). BAFF forms trimers and can further assemble into higher-order oligomers such as 60-mers. Computational modeling has suggested that this multimerization may critically regulate its biological activity and influence the clinical efficacy of targeted therapies (8). However, the pathological predominance of 60-mer BAFF in native diseased tissues has not been directly validated *in vivo*. This knowledge gap constrains the translational application of BAFF oligomeric forms in clinical settings. When BAFF binds to its receptors BAFF-R, TACI, and BCMA, it induces the formation of highly ordered clusters on lipid membranes. These clusters consist of receptors, ligands, and intracellular adaptors arranged in pentagonal or flattened hexagonal patterns. Such ordered clustering is essential for efficient activation of downstream signaling pathways (9).

BAFF expression is tightly regulated and widely distributed among various tissues, with the spleen showing the highest expression levels. Under both physiological and pathological conditions, multiple cell types can produce BAFF. Neutrophils in the spleen and bone marrow are the main constitutive producers, while conventional dendritic cells (cDCs) and monocyte subsets express lower levels (10). Upon infection or inflammatory stimulation, BAFF expression is significantly upregulated. During viral infection or bacterial lipoprotein stimulation, cDCs and neutrophils increase BAFF production via type I IFN signaling and the TLR2/MyD88/JNK pathway (10, 11). Under sustained inflammatory conditions, myeloid cells that produce BAFF undergo marked expansion. These include neutrophils, monocytes, and cDCs. The resulting elevated systemic load drives increased plasma cell differentiation and immunoglobulin secretion (12). Epithelial cells can also produce BAFF under specific stimuli. Respiratory syncytial virus infection stimulates BAFF production in airway epithelial cells via an IFN-β-dependent pathway. This response may promote local B-cell activation and antibody-mediated immunity (13).

BAFF expression is also finely regulated at the transcriptional and post-transcriptional levels. The BAFF gene has multiple alternative splicing variants, including the inhibitory isoform ΔBAFF and the BAFFX1 variant, the latter generating a premature stop codon due to partial intron retention. These splicing mechanisms add complexity to BAFF expression regulation (14). These mechanisms determine BAFF dynamics, though direct validation in diseased human tissues remains limited.

### 2.2 The three receptor systems of BAFF: BAFF-R, TACI, and BCMA

BAFF signals through three receptors that differ in expression, ligand preference, and functional output (**Table 2**).

**Table 2:** Comparative overview of BAFF receptors in physiological and pathological contexts.

Feature	BAFF-R	TACI	BCMA
Gene	TNFRSF13C	TNFRSF13B	TNFRSF17
Primary ligand(s)	BAFF	BAFF, APRIL	APRIL (high affinity), BAFF
Cellular expression	Mature and transitional B cells, subset of memory B cells; low on plasma cells (17)	Activated B cells, plasma cells; isoforms TACI-L and TACI-S (17)	Terminally differentiated plasma cells; frequently overexpressed on malignant plasma cells (27)
Key signaling pathways	Non-canonical NF-κB (DYRK1A–TRAF3–NIK); PI3K/AKT/mTOR (15, 16)	Classical and non-canonical NF-κB; context-dependent survival signaling	NF-κB; plasma cell survival and maintenance (27)
Physiological function	Mature B-cell homeostasis and survival; metabolic reprogramming; Tfh differentiation support (16, 37)	T-cell–independent immune responses; intestinal SIgA production; negative regulator of B-cell activation (22)	Long-lived plasma cell survival; redundant with APRIL/TACI axis (21)
Pathological role in autoimmunity	Lowers B-cell activation threshold; rescues autoreactive clones; drives Tfh differentiation (15, 37)	Context-dependent: haploinsufficiency attenuates humoral autoimmunity without compromising basal immunity; global KO may cause hyperactivation (23, 24)	Upregulated on activated B-cell subsets; correlates with plasmablast differentiation and autoantibody-secreting capacity (28)
Pathological role in cancer	Essential for survival of PCNSL, ALL, MCL, and CLL cells; drives pro-tumor gene programs (47–50)	Supports plasma cell survival in MM; decoy sTACI modulates local microenvironment (25, 52)	Highly expressed on malignant plasma cells in MM; key therapeutic target for CAR-T and bispecific antibodies (27)
Soluble/decoy form	None (membrane-bound only)	sTACI (generated by ADAM10 cleavage); sTACI-L shows higher inhibitory efficiency toward APRIL (25)	sBCMA (proteolytically shed); acts as ligand sink sequestering BAFF and APRIL (27)
Therapeutic relevance	Target for CAR-T and antibody therapy; prognostic biomarker in B-cell malignancies (98, 100)	Atacicept (soluble TACI-Fc decoy receptor): efficacy varies by disease context (effective in SLE, detrimental in MS) (109)	BCMA-directed CAR-T, bispecifics, and nanovesicle decoys; sBCMA serves as quantitative biomarker for tumor burden (54, 132, 138)

BAFF-R, B cell activating factor receptor; TACI, transmembrane activator and CAML interactor; BCMA, B cell maturation antigen; APRIL, a proliferation-inducing ligand; PCNSL, primary central nervous system lymphoma; ALL, acute lymphoblastic leukemia; MCL, mantle cell lymphoma; CLL, chronic lymphocytic leukemia; MM, multiple myeloma; SLE, systemic lupus erythematosus; MS, multiple sclerosis; sTACI, soluble TACI; sBCMA, soluble BCMA; SIgA, secretory IgA; Tfh, follicular helper T cell; KO, knockout.

Gene nomenclature for BAFF receptor family members (TNFRSF13C, TNFRSF13B, TNFRSF17) follows standard TNFSF receptor family conventions.

BAFF-R is the primary receptor for BAFF. It is expressed on mature, transitional, and memory B-cell subsets. BAFF-R signaling promotes B-cell survival through activation of the non-canonical NF-κB pathway. This pathway involves DYRK1A-mediated TRAF3 phosphorylation and stable NIK accumulation (15). BAFF-R can also activate the PI3K/AKT pathway to regulate metabolic reprogramming (16). On memory B cells, BAFF-R surface density is dynamically regulated. It directly sets the survival threshold and functional competence of these cells (17, 18). Receptor availability also gates the magnitude of downstream effector responses, as reduced expression limits B-cell output capacity (19). In addition, BAFF-R signaling indirectly affects the homeostasis of other immune cells, such as NK cells. However, it has no direct effect on NK-cell function (20).

TACI is encoded by TNFRSF13B. It can bind both BAFF and a proliferation-inducing ligand (APRIL). TACI mediates survival signaling for plasma cells (21). TACI signaling is critical for T-cell-independent (TI) immune responses. It is particularly important for the production of intestinal secretory IgA (SIgA) (22). TACI exerts context-dependent immunoregulatory effects and modulates plasma cell survival thresholds. Reduced TACI expression on activated B-cell subsets skews their differentiation trajectory away from antibody-secreting phenotypes (17). *In vivo*, TACI haploinsufficiency attenuates pathological humoral expansion while basal immunity remains intact (23). However, TACI functions in a context-dependent manner. Global knockout of TACI leads to B-cell hyperactivation and autoimmunity (24), illustrating a facet of this duality. TACI exists as two isoforms, TACI-L and TACI-S. Both can be cleaved by the metalloprotease ADAM10 to generate soluble forms (sTACI), which act as decoy receptors for BAFF and APRIL (25). This complexity provides a molecular basis for the dual roles of TACI in autoimmunity.

BCMA (encoded by TNFRSF17) binds APRIL with high affinity and also binds BAFF; it is a key receptor for maintaining plasma cell survival (21, 26). The high expression of BCMA on malignant plasma cells makes it an important therapeutic target. BCMA is selectively upregulated on terminally differentiated plasma cells and is frequently overexpressed in malignant plasma cell populations, forming the basis for targeted elimination strategies (27). On activated B-cell subsets, BCMA surface density correlates with plasmablast differentiation potential and autoantibody-secreting capacity (28). The soluble form sBCMA is generated by proteolytic shedding and functions as a ligand sink that competitively sequesters BAFF and APRIL, thereby modulating signal availability in the local microenvironment.

### 2.3 Core mechanisms and downstream effects of BAFF signaling pathways

The core mechanisms of BAFF signaling primarily depend on its specific binding to three receptors—BAFF-R, TACI, and BCMA—which subsequently activate a series of key downstream signal transduction cascades. Among these, the classical and non-canonical NF-κB pathways play critical roles (7). Binding of BAFF to BAFF-R promotes non-canonical NF-κB pathway activation through DYRK1A kinase. Specifically, DYRK1A phosphorylates TRAF3 at serine 29, interfering with its ability to mediate NIK degradation, leading to stable NIK accumulation and activation of the non-canonical NF-κB pathway (15).

In addition to the NF-κB pathway, BAFF signaling also activates the PI3K/AKT/mTOR pathway, which directly or indirectly regulates metabolic reprogramming of B cells (16). Studies have shown that in human naive B cells, BAFF-induced PI3K/AKT activation requires direct interaction of BAFF-R with BCR components CD79A and CD79B, and is enhanced by the AKT co-activator TCL1A. In contrast, switched memory B cells do not depend on BAFF-induced PI3K/AKT signaling for survival due to differences in surface BCR expression patterns and interactions (29). This differential activation of signaling pathways explains the differential dependence of various B-cell subsets on BAFF for survival.

BAFF signaling can also activate the NLRP3 inflammasome, participating in the regulation of B-cell inflammatory responses (30). Different oligomeric forms of BAFF differ in their ability to drive B-cell metabolic reprogramming (31). This signaling diversity underlies BAFF’s context-dependent outputs (**Table 2**; **Figure 1**).

## 3 BAFF as a core physiological “survival factor” for B cells

### 3.1 Regulation of B-cell development, maturation, and homeostasis

Based on the aforementioned receptor–signal transduction framework, the regulation of B-cell homeostasis by BAFF is manifested at multiple levels, including development, migration, survival, and antigen responses. In the bone marrow, the primary lymphoid organ for B cell development, BAFF directly induces CXCR5 expression in B-lineage cells, thereby influencing their migration to secondary lymphoid organs and exerting regulatory effects at an early stage of B-cell differentiation (32). This process is critical for establishing a complete B-cell repertoire. BAFF-deficient mice exhibit suppressed humoral immune responses, whereas BAFF overexpression leads to increased mature B-cell numbers and may result in autoimmune-like manifestations and B-cell lymphoproliferative disorders. These observations underscore the key role of BAFF in B-cell development and survival (1).

The regulation of B-cell homeostasis by BAFF is not only reflected in cell numbers but also profoundly affects the differentiation and function of B-cell subsets. Studies have shown that BAFF levels are closely associated with the distribution of circulating B-cell subsets. For example, in patients with autoimmune hepatitis, elevated BAFF levels positively correlate with the proportion of naive B cells and negatively correlate with class-switched and non-class-switched memory B cells, suggesting that BAFF may regulate immune responses by influencing B-cell differentiation stages (33).

BAFF can be produced by various myeloid cells such as neutrophils and dendritic cells, and these different sources of BAFF play unique and critical roles in antibody responses and autoimmune disease development (10, 12). In chronic graft-versus-host disease (cGVHD), pathologically elevated BAFF levels are associated with a high BAFF/B cell ratio and promote the persistence of BCR-activated B cells in peripheral blood and lesional tissues, thereby exacerbating disease manifestations (34). This reveals the disruptive impact of BAFF signaling on B-cell homeostasis under pathological conditions.

Naive B cells exhibit a significantly higher dependence on BAFF for survival than memory B cells, and this differential dependence constitutes a core feature of BAFF-mediated regulation of B cell subset homeostasis (29). Furthermore, BAFF enhances NOTCH signaling responsiveness and maintains SYK protein stability through BAFF-R, thereby lowering the threshold for B-cell activation and supporting the initiation of antigen responses (34). In the follicular B-cell compartment, homeostatic apoptosis limits cell lifespan to prevent compartmental atrophy due to intensified BAFF competition, further fine-tuning the size of the B-cell compartment (35).

BAFF also cooperates with APRIL to form a ligand–receptor regulatory network that synergistically supports B-lymphocyte survival and antibody responses; its local dysregulation is particularly prominent in renal autoimmune diseases such as membranous nephropathy and IgA nephropathy (36). In summary, BAFF precisely regulates B cell development and homeostasis, and its dysregulation is linked to immunopathological states.

### 3.2 Impact on B-cell functional differentiation and antibody production

As a critical survival factor for B cells, BAFF functions far beyond maintaining B-cell homeostasis; it profoundly influences the entire process of B cell development, differentiation, and antibody production. After entering the periphery, BAFF exerts even broader effects. It not only acts directly on B cells but also promotes the differentiation of Tfh cells through its receptor BAFF-R, a process dependent on NF-κB signaling (37). Tfh cells are essential for germinal center (GC) formation and the production of high-affinity antibodies; therefore, BAFF synergistically promotes GC responses and antibody production by acting simultaneously on B cells and Tfh cells (37). This mechanism is particularly prominent in autoimmune settings; for instance, in bullous pemphigoid, BAFF drives Tfh differentiation via BAFF-R–coupled PI3K/AKT signaling, thereby promoting autoantibody production (38).

The decisive influence of BAFF on B-cell differentiation fate is reflected in its fine-tuned regulation of distinct B-cell subsets. In an experimental kidney transplantation model, BAFF was required for the formation of intra-renal tertiary lymphoid organs (TLOs), promoting the formation of intra-renal B cell areas, expression of B-cell differentiation markers, and local plasma cell generation, thus driving local adaptive alloimmune responses (39). In Graves’ disease, high levels of thyroid hormones induce overexpression of BAFF in thyroid follicular cells and mononuclear cells, which in turn promotes the aberrant differentiation of CD11c^+^IgG^+^ autoreactive secretory B cells, aggravating disease relapse (40).

Furthermore, in the immunosuppressive microenvironment of cervical cancer, myeloid-derived suppressor cells induce B-cell differentiation into IL-10-producing B10 cells through the BAFF/BAFF-R pathway; the IL-10 secreted by B10 cells then activates STAT3 signaling in myeloid-derived suppressor cells (MDSCs), forming a positive feedback loop that promotes B10 cell differentiation (41). Collectively, these studies indicate that BAFF is a core regulator determining whether B cells differentiate toward effector, regulatory, or autoreactive fates.

In terms of antibody production, BAFF is indispensable. Inhibition of BAFF effectively reduces the production of anti-HLA.A2-specific IgG in a sensitized mouse model and suppresses B-cell differentiation and maturation in the bone marrow and spleen (42). The regulatory effects of BAFF and CD40L on B cell function differ: under conditions where anti-CD40 antibodies mimic CD40L, CD40L primarily promotes adaptive immune functions of B cells, whereas BAFF mainly enhances their innate immune functions (43). This difference stems from the differential regulation of NF-κB, ERK1/2, and PI3K-AKT signaling pathways by anti-CD40 and BAFF (43).

For the maintenance of long-lived plasma cells (i.e., memory plasma cells), BAFF and its homolog APRIL act as redundant survival mechanisms by binding to TACI and BCMA, providing a second essential survival signal that prevents endoplasmic reticulum stress-triggered caspase-12 activation. Together with integrin-mediated stromal cell contact signals, this ensures the sustained secretion of protective antibodies (44).

In neonates with sepsis, elevated BAFF levels promote the differentiation of peripheral blood B cells into CD19^+^CD24^hi^CD38^hi^ Bregs through the PI3K/AKT/mTOR pathway (45). In SLE patients, immune complex-driven neutrophil activation releases BAFF, and these neutrophils can directly promote B cell survival, proliferation, and differentiation into plasmablasts, thereby linking innate immune activation to pathological B cell responses (46). In summary, BAFF precisely regulates B-cell differentiation fate and antibody production through a multi-layer, multi-cellular complex network, which is both the basis for maintaining normal humoral immunity and the key link through which it participates in autoimmune diseases and tumor immune dysregulation. Thus, BAFF shifts from homeostasis maintainer to pathological driver once regulatory constraints are relieved in disease microenvironments.

## 4 Dual roles and mechanisms of BAFF in tumor immunity

BAFF’s functional output is not proposed to be intrinsically pro- or anti-tumorigenic. Rather, it is proposed to emerge from the interplay of four variables: ligand concentration, oligomeric form, cellular source, and receptor availability. **Table 1** maps how these variables configure across hematological malignancies, solid tumors, and autoimmune diseases. In the following sections, we examine the evidence for each variable, treating autoimmune diseases not as a separate silo but as a conceptual test case for the same proposed framework, recognizing that cross-disease isomorphism does not constitute experimental proof.

### 4.1 The high-concentration, chronic wing: BAFF supports malignant B cell survival and immunosuppression

BAFF and its receptor system play central roles in the development, progression, and drug resistance of various B-cell malignancies. Its pro-tumor effects are not only reflected in directly supporting the survival and proliferation of malignant B cells but also in profoundly shaping the immunosuppressive tumor microenvironment. In multiple B-cell malignancy models, BAFF-R signaling contributes to tumor progression. BAFF-R signaling drives survival and chemoresistance across multiple B-cell malignancies, including primary central nervous system lymphoma (PCNSL), acute lymphoblastic leukemia (ALL), mantle cell lymphoma (MCL), and chronic lymphocytic leukemia (CLL) (47–50). In CLL, BAFF binding to BAFF-R is specifically required for activating pro-tumor gene programs that enable peripheral dissemination, rather than merely sustaining survival (50).

In addition, intercellular communication within the tumor microenvironment amplifies the pro-survival effects of BAFF. Recombinant BAFF (rhBAFF) protects MCL cells from cytarabine-induced apoptosis, suggesting that high BAFF concentrations in the microenvironment constitute a chemoresistance factor (49). MCL cells polarize monocytes into specific MCL-associated macrophages by secreting IL-32β; these macrophages in turn support tumor survival mainly through BAFF-driven soluble factor-mediated intercellular communication, forming a pro-survival circuit driven by the IL32β/BAFF axis (51). In multiple myeloma (MM), BAFF and its homolog APRIL play important roles in the survival and progression of malignant plasma cells by binding to BCMA and TACI (52). In the bone marrow microenvironment, a neutrophil–stromal cell positive feedback loop establishes a self-sustaining BAFF-rich niche that promotes malignant plasma cell survival independently of systemic therapy (53). This localized microenvironmental persistence explains why tumor-intrinsic BAFF signaling may evade systemic ligand depletion. Accordingly, multiple therapeutic strategies targeting this system are under active investigation, engineered cellular membrane-based vesicles displaying reconstructed BCMA can act as decoy proteins to capture excess APRIL and BAFF in the microenvironment, thereby inhibiting MM cell survival and synergizing with bortezomib (54).

The pro-tumor effects of BAFF are not limited to hematological malignancies; its ability to mediate immune evasion in solid tumors has also attracted considerable attention. In solid tumors such as triple-negative breast cancer, tumor cell overexpression of BAFF promotes tumor growth *in vivo* by inducing IL-10-secreting CD5^+^ Bregs, which in turn suppress anti-tumor CD8^+^ T-cell responses (55). In hepatocellular carcinoma, the BAFF/NF-κB axis is activated in cancer-associated fibroblasts, promoting the malignant phenotype and chemoresistance of co-cultured Huh7 cells (56). In colorectal cancer, BAFF levels in tumor tissues are significantly elevated and positively correlate with IL-8; its expression is negatively correlated with the degree of immune infiltration, suggesting its involvement in shaping a suppressive tumor microenvironment (57).

Even in some seemingly paradoxical solid tumor models, the systemic impact of BAFF tends to be overall pro-tumor. Taking melanoma as an example, although BAFF overexpression can locally delay primary tumor growth by modulating monocytes to reduce their immunosuppressive phenotype, chronically elevated systemic BAFF simultaneously impairs T-cell-mediated systemic anti-tumor responses and reduces sensitivity to PD-1 blockade therapy (58). This model reveals that BAFF may play entirely different roles locally versus systemically. In a Helicobacter felis-infected NLRC5-deficient mouse model of gastric mucosa-associated lymphoid tissue (MALT) lymphoma, gastric BAFF production was significantly increased, and blocking BAFF signaling markedly reduced MALT formation (59). Collectively, these model-specific findings support BAFF as a candidate contributor to immune evasion in the cited tumor models.

Yet the same capacity to remodel local immune niches — whether by recruiting regulatory B cells or sustaining ectopic lymphoid structures — operates with striking fidelity in autoimmune microenvironments, where the net outcome shifts from immune evasion to tolerance breakdown. The survival-licensing function of BAFF-R described here is not unique to malignancy. Autoreactive B cells in systemic autoimmunity exploit the identical signaling module to escape peripheral deletion, where chronically elevated systemic BAFF breaks tolerance by rescuing clones that would otherwise be eliminated. Whether the recipient cell is a malignant lymphocyte or an autoreactive one, the signaling output is the same; only the immunological context differs.

### 4.2 The moderate, transient window: BAFF is proposed to sustain tumor-infiltrating B cells and tertiary lymphoid structures

We now review the more limited evidence for BAFF-mediated anti-tumor effects, predominantly from melanoma models. BAFF shows context-dependent immunomodulatory activity in specific tumor types and microenvironments. BAFF is proposed to support the maintenance of tumor-infiltrating B cells (TIBs) and adaptive immunity, based on the cited melanoma models. Substantial evidence indicates that TIBs are associated with favorable prognosis in melanoma, breast cancer, lung cancer, and colorectal cancer, where they participate in anti-tumor immunity through antibody production, antigen presentation, and cytokine secretion (60–63). As a survival signal for mature B cells, BAFF may maintain basal TIB survival through BAFF-R-mediated non-canonical NF-kB activation, potentially supporting their persistence within the tumor microenvironment. In melanoma models, BAFF overexpression upregulated B-cell costimulatory molecules, enhanced B-cell antigen presentation to CD4-positive T helper cells, and promoted Th1 polarization and memory T-cell formation (64). BAFF is proposed to provide a survival signal that may help maintain the B-cell zone within TLS and may support local GC reactions, somatic hypermutation, and the production of high-affinity tumor-reactive antibodies (60, 65). TLS are ectopic lymphoid aggregates formed in non-lymphoid tissues under chronic inflammation, including tumors, and their function depends on coordinated interactions among local immune cells (63, 66). Direct evidence that BAFF causes tumor-associated TLS formation in human tumors is lacking. The available evidence for a requirement for BAFF in tertiary lymphoid organ formation comes from experimental kidney-transplant models, not tumor systems (39).

Furthermore, BAFF can activate multidimensional immune responses by remodeling myeloid cells. In melanoma models, BAFF overexpression was observed to reduce PD-L1 expression and alleviate the immunosuppressive phenotype of tumor-infiltrating monocytes, thereby locally delaying primary tumor growth (58). However, the same study found that chronically elevated systemic BAFF impairs T-cell-mediated systemic anti-tumor responses and reduces sensitivity to PD-1 blockade. Therefore, the role of BAFF in this model cannot be simplistically categorized as “anti-tumor”; its net effect depends on the level of evaluation, such as local primary tumor growth rate versus systemic immune surveillance and immunotherapy response.

Meanwhile, tumor-associated neutrophils can drive B-cell differentiation into plasma cells through membrane-bound BAFF, leading to local IgG production, which may mediate antibody-dependent cell-mediated cytotoxicity (ADCC) against tumor cells (67). Whether this neutrophil–BAFF–IgG axis promotes anti-tumor immunity via ADCC or leads to immune dysregulation in certain complex contexts remains to be clarified by future studies, but it clearly demonstrates the potential pathway through which BAFF can enhance anti-tumor humoral responses. Since BAFF receptors (BAFF-R, BCMA, and TACI) are selectively expressed on malignant B cells but not on early B cell progenitors, this provides a theoretical basis for designing BAFF ligand-based immunotherapies that exploit rather than simply block BAFF biology for anti-tumor purposes. BAFF ligand-based CAR-T that can simultaneously target all three receptors have shown potent cytotoxicity against multiple B-cell malignancies in preclinical models while minimizing off-target toxicity to normal hematopoietic progenitors (68).

Overall, anti-tumor evidence remains limited compared to pro-tumor data, requiring further validation. This asymmetry notwithstanding, the tumor evidence reveals a fundamental principle: BAFF is not intrinsically pro- or anti-tumorigenic, but context-dependent. This capacity of BAFF to sustain organized B cell zones and promote antigen presentation locally mirrors its role in ectopic lymphoid neogenesis at sites of chronic inflammation. In both settings, the net outcome depends on whether the local antigenic drive is tumor-directed or self-directed, and whether BAFF elevation is transient or sustained.

### 4.3 The “Inverted U-Shaped” concentration-effect hypothesis: a multi-factor framework for solid tumors

BAFF exhibits paradoxical pro-tumor and anti-tumor activities in solid tumors. This apparent “paradox” is not irreconcilable but depends on the dynamic interplay of multidimensional variables, including molecular multimeric forms, cellular targeting heterogeneity, spatiotemporal anatomical sites, and cytokine networks. Based on available evidence, this review proposes a systematic multi-factor explanatory framework to provide testable hypotheses for future research. The source, molecular form, and local concentration of BAFF within the tumor microenvironment are proposed variables that may shape its net effect. BAFF can be produced by tumor cells themselves, cancer-associated fibroblasts, and various myeloid cells (1, 56); these different sources differ fundamentally in spatial distribution and oligomerization status. Membrane-bound BAFF or 60-mer higher-order oligomers tend to strongly activate the canonical NF-κB pathway (31), whereas soluble trimers mainly sustain basal non-canonical signaling (15). When membrane-bound or 60-mer BAFF is proposed to predominate in the tumor microenvironment, it may be more prone to drive aberrant B cell survival and elicit pro-tumor chronic inflammation; conversely, low-concentration soluble BAFF may tend to merely sustain basal homeostasis of TIBs, favoring local anti-tumor immunity.

The target cell types of BAFF and their receptor expression profiles are also proposed variables that may shape the final outcome. In solid tumors, BAFF indirectly remodels the microenvironment mainly by modulating immune cells, and it exhibits significant bidirectional differentiation potential at the B-cell level. In a cervical cancer model, BAFF induced differentiation of CD5^+^ B cells into IL-10^+^ Bregs through the BAFF-R axis, forming a positive feedback loop with MDSCs that suppressed anti-tumor T-cell responses (41); however, in the melanoma environment, BAFF also upregulated B-cell costimulatory molecules, enhanced antigen presentation to CD4^+^ T cells, and promoted Th1 polarization (64). At the monocyte/macrophage level, BAFF also has dual effects: locally in melanoma, it can downregulate PD-L1 on monocytes and alleviate their immunosuppressive phenotype to delay primary tumor growth; however, the peripheral immune tolerance induced by its systemic elevation often “overrides” this local benefit (58).

To more dynamically integrate the above variables, this review proposes a testable “inverted U-shaped” concentration-effect relationship (**Figure 2**). No established concentration thresholds currently define low, intermediate, or high BAFF exposure. The three zones are conceptual labels anchored to relative biological contexts, namely basal homeostatic levels, transient local upregulation, and chronic or systemic elevation, not standardized concentration cutoffs. Direct low-to-intermediate-to-high titration in a single system has not been reported. At very low concentrations, BAFF may be insufficient to sustain effective local humoral immunity; moderate concentrations (such as transient local upregulation) are theoretically compatible with TLS maintenance and local anti-tumor responses; however, excessively high or chronically systemic elevated BAFF is associated with regulatory B cell expansion, T-cell exhaustion, and tumor immune evasion. This concentration-dependent interpretive framework may provide a conceptual reference for understanding clinical observations: the association between short-term local BAFF upregulation and better prognosis, and the correlation between persistently high serum BAFF levels and resistance to immune checkpoint blockade. This framework remains an integrative working hypothesis, pending direct validation by oligomer-specific assays. Nevertheless, its explanatory logic may extend beyond oncology. This logic posits concentration, oligomeric form, and cellular source as proposed variables that may jointly shape functional output.

Limitations of the proposed framework. It is critical to emphasize that no cited study has performed a complete low-to-intermediate-to-high BAFF titration in the same experimental system while holding oligomeric form, cellular source, and receptor availability constant. The inverted U-shaped model therefore remains a working hypothesis integrating existing fragmentary observations. Local (tissue microenvironment) and systemic (serum) BAFF concentrations are not interchangeable variables, and their discordance is not captured by the current schematic. Future studies employing conditional gene-modified models, spatial transcriptomics, and oligomer-specific detection assays will be required to test the predictions advanced here.

In the following section, we apply this framework to autoimmune diseases as an external, conceptually supportive test case, examining whether the same proposed variables may influence BAFF’s pathogenic heterogeneity across the cancer-autoimmunity spectrum.

### 4.4 Translating the framework to autoimmunity: hypotheses and testable predictions

The four-variable framework developed above can be reverse-mapped to autoimmunity as a set of falsifiable predictions. To avoid duplication, this section is deliberately confined to the framework-level logic and the predictions it generates; the mechanistic evidence underlying each disease configuration is examined in detail in the disease-specific discussion that follows. If the parallelism holds, ligand concentration, oligomeric form, cellular source, and receptor availability are predicted to influence BAFF’s net effect in autoimmunity in parallel with the proposed oncology model. Three configurations illustrate this mapping. Systemic lupus erythematosus exemplifies a high-concentration, systemically distributed configuration corresponding to the right wing of the curve (46). Primary Sjögren’s syndrome (pSS) exemplifies a tissue-restricted, high-concentration configuration in which ligand elevation is confined to the target organ (69). Neuromyelitis optica spectrum disorder (NMOSD) exemplifies a configuration in which a single cellular source dominates BAFF production, isolating the source variable from concentration effects (70). These configurations are analogical, not experimentally validated, and their value lies in three testable predictions: (i) moderate, transient BAFF elevation in defined tissue niches may support protective humoral immunity, whereas sustained or systemic elevations may contribute to pathology; (ii) the oligomeric state of BAFF, rather than total BAFF level alone, may determine pathogenic potential (31); and (iii) therapeutic design should consider local BAFF sources and the preservation of beneficial BAFF-mediated functions, not only systemic ligand neutralization.

It is important to note that the role of BAFF in regulatory B-cell induction remains debated. While murine studies suggest that BAFF promotes IL-10-producing B cells that suppress inflammation (71), human data indicate that BAFF-driven Breg expansion may be functionally unstable or insufficient to overcome overwhelming effector B-cell activation (72). Notably, APRIL—a close homolog of BAFF—has also been shown to induce IgA^+^ IL-10-producing Bregs with potent immunosuppressive functions (73), further highlighting the complexity of the BAFF/APRIL system in regulating Breg biology across species and experimental systems. This human–mouse divergence implies that the Breg-expansion arm of the high-BAFF model may be less stable in human disease than in murine systems, a prediction testable by oligomer-specific stratification of patient cohorts.

## 5 Autoimmune diseases as a conceptual testbed for the cross-disease BAFF framework

Building on the framework-level configurations outlined above, this section examines the disease-specific mechanistic evidence for each variable. If BAFF’s functional duality is proposed to be jointly influenced by concentration, oligomeric form, cellular source, and receptor availability, then autoimmune diseases provide an external, conceptually supportive test case for this proposition. SLE, pSS, and NMOSD exemplify how the same four variables—operating in different configurations—drive tolerance breakdown rather than tumor immune evasion.

### 5.1 BAFF as a threshold-regulator of B-cell tolerance: from central deletion to peripheral escape

The inverted U-shaped framework developed above proposes that ligand concentration is a variable that may shape functional output. Sustained, systemic BAFF elevation — the right wing of the curve — offers a paradigmatic test case. In autoimmune diseases, overexpression of BAFF breaks the physiological threshold of B cell survival signals, driving autoreactive B cells to escape tolerance elimination. In healthy individuals, B-cell tolerance is maintained by multiple checkpoints: central deletion in the bone marrow, receptor editing, and peripheral anergy. BAFF functions as a dose-sensitive regulator that sets the stringency of peripheral tolerance: when BAFF levels are low, autoreactive B cells with intermediate BCR affinity fail to compete for survival signals and are eliminated; when BAFF levels are elevated, these otherwise eliminated clones are rescued, breaching the tolerance threshold (1).

In SLE, serum BAFF levels are closely associated with disease activity. They correlate positively with anti-dsDNA antibody titers and negatively with complement C3 levels (46). *In vitro* experiments confirm that BAFF stimulation upregulates surface BAFF-R, BCMA, and TACI expression on normal B cells, thereby lowering the activation threshold and driving differentiation, proliferation, and survival (3). These effects are reinforced by BAFF-dependent priming of the NLRP3 inflammasome, which couples B cell survival signals to inflammatory cytokine output in a BCR-dependent manner (30). This indicates that BAFF functions beyond a mere survival factor; it can integrate other signals to finely regulate B-cell inflammatory responses and fate.

In specific tissue microenvironments, BAFF production and B cell aberrant activation form a vicious cycle. In ectopic lymphoid tissues formed in nasal polyps, BAFF levels are significantly elevated, and the number of BAFF-positive cells is negatively correlated with the frequency of B cell apoptosis, indicating that BAFF promotes the formation and maintenance of ectopic lymphoid tissues by inhibiting B cell apoptosis (74). In cGVHD, pathologically elevated BAFF enhances BCR responsiveness and promotes autoantibody production by upregulating B cell NOTCH2 expression (34). In the proposed framework, SLE represents a relative high-concentration, systemic, myeloid-source configuration, in which persistently elevated soluble BAFF contributes to pathological B cell expansion beyond the tolerance threshold.

### 5.2 BAFF-mediated T-cell reprogramming: Tfh, Treg, and the autoimmune-tumor paradox

A second variable in the framework concerns target-cell receptor availability and its downstream fate. In the tumor microenvironment, BAFF receptor density on B cells and monocytes is proposed to gate whether signaling yields immune activation or suppression; an analogous receptor-availability logic operates in autoimmune settings. BAFF directly affects the development and function of T-cell subsets, adding another layer to its immunoregulatory repertoire. Studies have shown that BAFF deficiency (but not APRIL deficiency) significantly suppresses Tfh development, GC formation, and antigen-specific antibody production (37). BAFF directly promotes the expression of Tfh-characteristic genes through BAFF-R, a process dependent on NF-κB signaling and requiring BAFF-R expression on T cells (37). In bullous pemphigoid (BP), BAFF mediates the differentiation of naive CD4^+^ T cells into Tfh cells through the BAFF-R–PI3K/AKT–mTOR pathway; anti-BAFF monoclonal antibody treatment reduces the proportion of Tfh cells and autoantibody production *in vivo* (38).

BAFF also regulates Tregs, though the direction of regulation appears disease-specific. In C57BL/6 mouse models, mice expressing a BAFF transgene had abnormally elevated Treg proportions, whereas BAFF-deficient mice had significantly reduced Tregs (75). In SLE patients, BAFF blockade with belimumab significantly increases the proportion of functional Tregs (76); whereas in acute GVHD, BAFF directly reduces the proportion of Tregs (77). This bidirectional regulation suggests that the BAFF–Treg relationship is context-dependent—varying with the underlying inflammatory milieu and the presence of other cytokines—rather than a fixed biological relationship.

In chronic obstructive pulmonary disease (COPD), BAFF expression is increased and can suppress CD8^+^ T-cell apoptosis while increasing IFN-γ expression in CD4^+^ T cells and perforin and granzyme B expression in CD8^+^ T cells, thereby potentially participating in inflammatory responses (78). In patients with biliary atresia, BAFF levels positively correlate with IFN-γ levels; BAFF inhibition reduces portal inflammation and IFN-γ expression in T cells while expanding Tregs (79).

### 5.3 Organ-specific BAFF sources and ectopic lymphoid neogenesis

The pathogenic patterns of BAFF exhibit remarkable disease specificity and are proposed to be shaped by the cellular source of BAFF and the local microenvironment in which it acts. A perspective often underappreciated in autoimmune-focused reviews: where BAFF comes from is as important as how much BAFF is present. In SLE, BAFF is systemically elevated, derived primarily from myeloid cells (neutrophils, monocytes, dendritic cells) responding to type I IFN and TLR signals (12, 80). Immune complex-driven neutrophil activation releases BAFF, which directly promotes B cell survival, proliferation, and differentiation into plasmablasts, linking innate immune activation to pathological B cell responses (46). This systemic source accounts for the widespread B cell hyperactivity characteristic of SLE.

In pSS, by contrast, BAFF is produced locally by salivary gland epithelial cells, plasmacytoid dendritic cells, and monocytes within the inflamed gland (69, 81). Salivary gland epithelial cells produce large amounts of BAFF in response to pro-inflammatory cytokines, creating a high-concentration niche that promotes B cell recruitment, interactions between T cells and B cells, and ectopic GC formation (69). This local source may help explain the organ-specific nature of pSS pathology, with BAFF acting as a candidate contributor to ectopic lymphoid neogenesis within the target organ.

In NMOSD, a third distinct source has been identified: aberrant bone marrow granulopoiesis generates ISG15^+^ neutrophils that produce large amounts of BAFF, sufficient to drive the maturation of antibody-secreting cells (ASCs) and autoantibody production in the cited experimental system (70). This bone marrow-derived source accounts for the systemic B-cell activation observed in NMOSD, linking hematopoietic dysregulation to autoimmune pathology. In CD14^+^ monocytes from NMOSD patients, the long non-coding RNA MALAT1 upregulates BAFF expression by sponging miR-30b-5p, thereby triggering B cell autoimmune responses (82).

In RA, BAFF is one of the key pro-inflammatory cytokines in the inflamed synovium. Liposome-delivered dexamethasone exerts anti-inflammatory effects partly by downregulating BAFF (83). In interstitial pneumonia with autoimmune features (IPAF), serum BAFF and IL-17 levels are elevated; combined detection may help distinguish IPAF from other types of interstitial lung disease (84). In autoimmune pulmonary alveolar proteinosis (APAP), serum and bronchoalveolar lavage fluid levels of BAFF and APRIL are significantly elevated, and serum BAFF levels correlate with disease severity and pulmonary function parameters (85).

This diversity of cellular sources and differences in local microenvironments may jointly shape the distinct pathogenic mechanisms of BAFF across autoimmune diseases. These source-specific patterns mirror the tumor microenvironment, where BAFF from CAFs versus neutrophils likewise yields divergent outcomes—a cross-disease parallelism that supports further evaluation of the cellular-source variable as a potential predictive axis across the cancer–autoimmunity spectrum.

## 6 Therapeutic strategies and application prospects targeting the BAFF/BAFF-R axis

Therapeutic targeting of the BAFF axis may be informed by the same proposed concentration–form–context variables that may shape its functional output, rather than by disease category alone. The current landscape of BAFF-targeted therapeutics is summarized in **Table 3**.

**Table 3:** BAFF-targeted therapeutics in autoimmune diseases and B-cell malignancies.

Agent	Target	Mechanism of action	Key indications	Development stage	Key clinical/preclinical findings	Reference
Belimumab	Soluble BAFF	Human anti-BAFF monoclonal antibody; computational modeling suggests that it interferes with BAFF 60-mer formation, a mechanistic distinction from tabalumab that may contribute to the divergent clinical outcomes observed with the two agents; no head-to-head trial has directly compared them	SLE, lupus nephritis	Approved (FDA/EMA)	Reduces disease activity and autoantibody titers; delays organ damage; favorable long-term safety profile	(8, 87–92, 113)
Tabalumab	Soluble BAFF	Anti-BAFF mAb; distinct binding mode from belimumab	SLE, MM	Discontinued	In silico analysis suggests it fails to interfere with BAFF 60-mer formation, offering a potential mechanistic basis for its lack of demonstrated clinical efficacy (in silico analysis only; no head-to-head trial); no significant benefit in MM	(1, 8)
Telitacicept	BAFF + APRIL	Recombinant TACI-Fc fusion protein (dual inhibitor)	SLE,RA, IgAN, MG, Sjögren’s disease	Approved (China)^1^	Favorable efficacy signals vs belimumab in a non-head-to-head real-world SLE study; Phase III TELIGAN trial demonstrated significant proteinuria reduction in IgAN; subsequently approved in China for IgAN and Sjögren’s disease^1^	(95, 96)
Atacicept	BAFF + APRIL	Soluble TACI-Fc decoy receptor neutralizing BAFF and APRIL	MS, IgAN	Terminated (MS); Approved (FDA, 2026 for IgAN)^2^	unexpectedly exacerbated disease activity in MS, highlighting context-dependent BAFF/APRIL neutralization effects	(109, 110)
Povetacicept (ALPN-303)	BAFF + APRIL	Affinity-enhanced TACI-Fc via directed evolution	IgAN, membranous nephropathy	Phase III (IgAN)	Affinity-enhanced dual BAFF/APRIL antagonist via directed evolution; stronger B cell suppression and autoantibody reduction than earlier-generation inhibitors (preclinical/indirect comparison); good tolerability	(126–128)
Ianalumab (VAY736)	BAFF-R	Afucosylated anti-BAFF-R mAb with enhanced ADCC	ITP, pSS	Phase III (ITP, pSS)	combines BAFF-R targeting with enhanced ADCC-mediated B-cell depletion	(102–104)
BAFF-R CAR-T	BAFF-R	Chimeric antigen receptor T cells targeting BAFF-R	CLL, ALL	Phase I (NCT05370430, NCT04690595)	Cytotoxic in CD19-negative/resistant models	(98–101, 131, 132)
BAFF ligand-based CAR-T	BAFF-R/BCMA/TACI	Ligand-based CAR simultaneously targeting all three receptors	MCL, MM, ALL	Preclinical	Potent cytotoxicity across heterogeneous B-cell tumors; minimizes risk of single-antigen escape	(68)
Bispecific BAFF-R/BCMA CAR-T	BAFF-R + BCMA	Dual-target CAR-T	RRMM	Preclinical	Controls heterogeneous plasma cell populations; compensates for BCMA downregulation and antigen escape	(132)
BAFF-based trifunctional TCE	BAFF-R/BCMA/TACI	Anti-CD3 Fab fused to BAFF ligand bridging T cells and tumor cells	B-cell malignancies	Preclinical	Triggers robust tumor immunity; efficacy superior to blinatumomab in preclinical leukemia models only; no clinical comparison	(133)

BAFF, B cell activating factor; APRIL, a proliferation-inducing ligand; BAFF-R, BAFF receptor; BCMA, B cell maturation antigen; TACI, transmembrane activator and CAML interactor; SLE, systemic lupus erythematosus; IgAN, IgA nephropathy; MG, myasthenia gravis; MS, multiple sclerosis; ITP, immune thrombocytopenia; MM, multiple myeloma; CLL, chronic lymphocytic leukemia; MCL, mantle cell lymphoma; ALL, acute lymphoblastic leukemia; RRMM, relapsed/refractory multiple myeloma; ADCC, antibody-dependent cellular cytotoxicity; TCE, T-cell engager; FDA, U.S. Food and Drug Administration; EMA, European Medicines Agency. References are cited as numbered references in the manuscript bibliography.

### 6.1 Existing targeted agents and their clinical efficacy

The systematic dissection of the BAFF signaling network and its roles in B-cell homeostasis, tumor immunity, and autoimmune pathology described above indicates that the BAFF/BAFF-R axis constitutes a therapeutic node with high intervention potential (**Figure 3**). Based on this, drugs targeting BAFF and its receptors have become important strategies for treating autoimmune diseases and B-cell malignancies, and their clinical efficacy has been validated in multiple diseases. Preclinical studies have provided proof-of-concept for BAFF pathway targeting. In the spontaneous lupus mouse model (MRL/lpr), treatment with a novel BAFF antagonist BAFF-Trap (fusing TACI and BAFF-R binding domains) significantly reduced serum autoantibody levels, decreased renal IgG deposition, improved proteinuria, and increased mouse survival (86).

**Figure 3:**
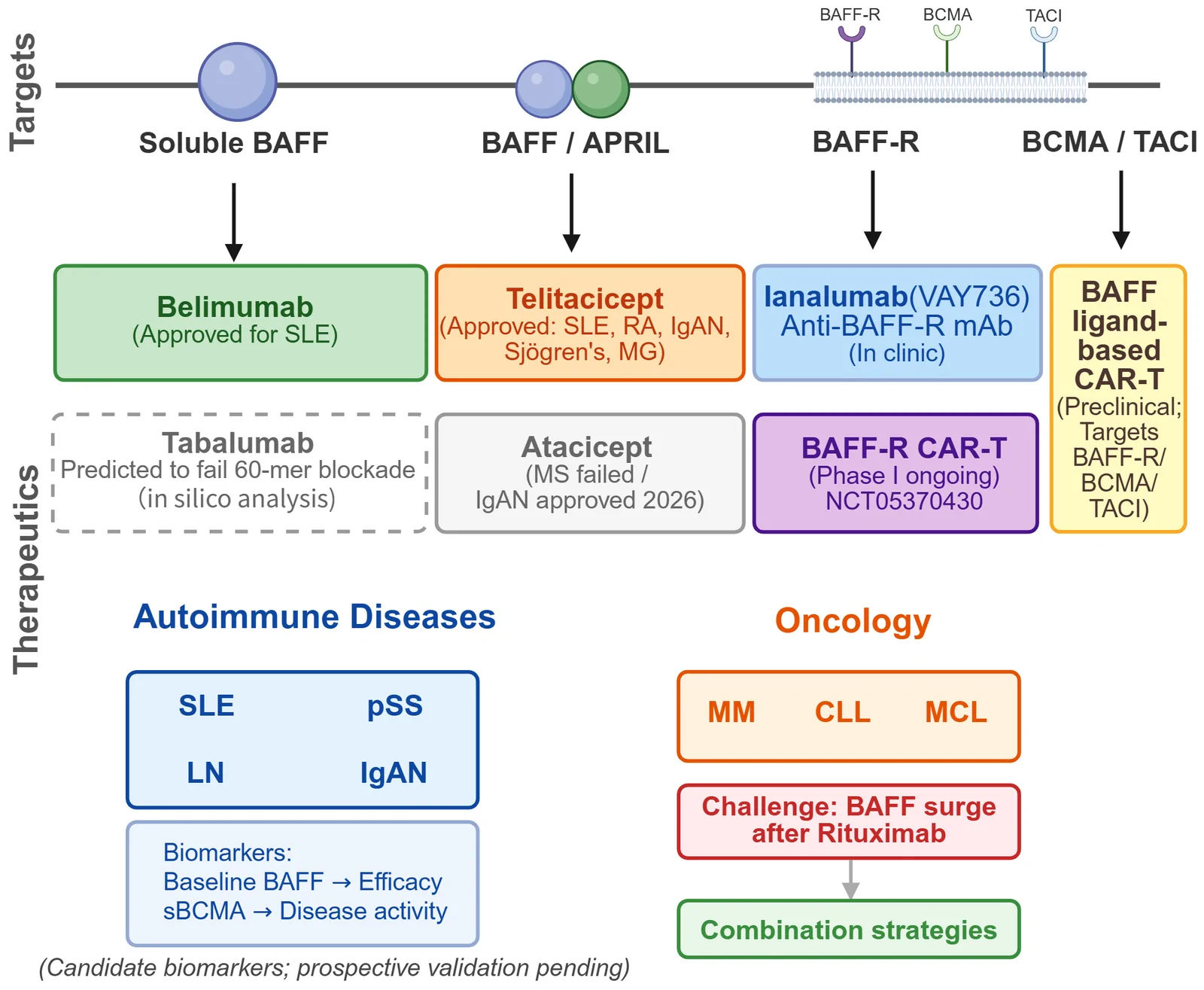
(Top) Interventions are categorized by molecular target: soluble BAFF (belimumab, approved for SLE; tabalumab, shown with a dashed border to indicate discontinued status); BAFF and APRIL (telitacicept, approved in China for SLE, RA, IgAN, Sjögren’s disease, and MG; atacicept, which failed in MS trials but received FDA approval for IgAN in 2026 as the first FDA-approved BAFF/APRIL-targeting medicine); and receptors (ianalumab/VAY736, an anti-BAFF-R monoclonal antibody in clinical development; BAFF-R CAR-T in Phase I trials, NCT05370430; preclinical BAFF ligand-based CAR-T engaging BAFF-R, BCMA, and TACI). The tabalumab annotation “predicted to fail 60-mer blockade” derives from in silico analysis (8) and is not based on direct biochemical evidence. (Middle) Disease applications span autoimmune conditions (SLE, pSS, LN, IgAN) and oncology (MM, CLL, MCL). The challenge of BAFF surge following rituximab-mediated B-cell depletion and the potential of combination strategies are highlighted. (Bottom) Biomarker-driven stratification: baseline BAFF as a candidate predictor of treatment response and sBCMA as a candidate readout of disease activity. These are associative candidate biomarkers, not validated selection rules; prospective validation is pending. Arrows in **Figure 3** indicate target–agent–disease relationships only and do not encode evidence levels. Created in BioRender. Shang, J. (2026) https://BioRender.com/0gqtqxi.

In the autoimmune field, belimumab, as the first targeted biologic approved for SLE, has had its efficacy and safety extensively confirmed (87). Multiple randomized controlled trials have shown that belimumab in combination with standard therapy effectively reduces SLE disease activity, decreases glucocorticoid dosage, and delays organ damage (88). At the cellular level, BAFF inhibitor belimumab reduces disease activity in SLE, and its efficacy correlates with dynamic changes in circulating B-cell compartments (89). A large integrated analysis further confirmed its safety in adult SLE patients, with adverse event rates similar to placebo (90). Moreover, belimumab has shown potential in pediatric SLE to suppress disease activity, reduce glucocorticoid and immunosuppressant use, and delay renal damage (91); in severe lupus nephritis patients requiring dialysis, belimumab also demonstrated therapeutic value in improving renal and immunological parameters and reducing disease activity (92). Beyond SLE, the BAFF target of belimumab is also highly expressed in Sjögren’s disease (SjD) and participates in pathogenesis, suggesting potential therapeutic value of anti-BAFF strategies in SjD (93). Animal model studies have shown that BAFF-blocking monoclonal antibodies significantly reduce salivary gland lymphocyte infiltration and improve salivary flow rates, providing preliminary evidence for clinical translation of this strategy (93). Another anti-BAFF antibody, tabalumab, has also been studied; in silico analysis suggests that its binding mode to BAFF differs from that of belimumab and may fail to interfere with BAFF 60-mer formation (8), offering a potential mechanistic basis for its lack of demonstrated clinical efficacy. No head-to-head randomized trial has compared the two agents, and differences in trial populations, endpoints, and development programs preclude any direct comparative-efficacy conclusion. In addition, the dual BAFF/APRIL inhibitor telitacicept (94) showed more favorable clinical signals than belimumab comparators in an observational, non-head-to-head real-world study (95), and significantly reduced proteinuria in IgA nephropathy in the Phase III TELIGAN trial (96). The real-world comparison was not randomized or head-to-head and does not establish superior efficacy. Moreover, telitacicept has been approved in China for multiple indications, including SLE, RA, and generalized myasthenia gravis (gMG)^1^; the Phase III TELIGAN trial subsequently demonstrated significant proteinuria reduction in IgA nephropathy (96), leading to regulatory approval for IgAN and Sjögren’s disease in 2026^1^. In MG patients, those with high baseline neutrophil levels benefited more from telitacicept, providing clinical evidence for the potential of this class of drugs in MG treatment (97).

In the field of B-cell malignancies, strategies targeting the BAFF pathway have also attracted considerable attention. Although tabalumab showed no significant efficacy in a phase II study of relapsed/refractory multiple myeloma (RRMM) (1), emerging immunotherapies such as BAFF-R-targeted CAR-T cells (e.g., PMB-CT01) have entered Phase I clinical trials (NCT05370430, NCT04690595) (98, 99). Preclinical validation confirmed robust anti-tumor activity of BAFF-R CAR-T, supporting its promising therapeutic potential for B-cell malignancies (100). Moreover, through “CAR Pooling” screening, it was found that incorporating the BAFF-R signaling domain into CAR designs enhances CAR-T cell cytotoxicity under prolonged antigen stimulation, providing new ideas for next-generation immunotherapies (101).

In other autoimmune diseases, BAFF-targeted therapy also shows application value. For example, in primary immune thrombocytopenia (ITP), spleen and serum BAFF levels are elevated. The BAFF-R-targeting agent ianalumab (VAY736) is in Phase III clinical development for primary immune thrombocytopenia (VAYHIT2, NCT05653219) (102, 103) and primary Sjögren’s syndrome (NEPTUNUS-1, NCT05350072; NEPTUNUS-2, NCT05349214) (104). In MG, low-dose rituximab treatment leads to elevated serum BAFF levels, and BAFF levels negatively correlate with clinical severity (105). Furthermore, in MuSK antibody-positive MG patients treated with ofatumumab, decreased BAFF levels were observed and correlated with better clinical outcomes (106). In neuropsychiatric SLE (NPSLE), elevated serum BAFF levels correlate with disease activity and poor prognosis, suggesting its potential as an auxiliary diagnostic and prognostic biomarker (107). Similarly, cerebrospinal fluid BAFF levels are significantly elevated in neurosarcoidosis (NS) patients and decrease after treatment, indicating its utility as a quantitative biomarker for this disease (108).

Collectively, these studies indicate that targeting the BAFF pathway has diverse therapeutic potential across different disease contexts, and its efficacy is closely related to disease type, drug mechanism of action, and patient immune status.

### 6.2 Challenges, safety, and combination strategies of targeted therapy

Although therapeutic strategies targeting the BAFF/APRIL axis have shown significant efficacy in various diseases, their clinical application still faces numerous challenges and safety considerations. The foremost challenge is the heterogeneity of treatment responses, which necessitates the development of precision biomarkers to guide patient stratification. The divergent outcomes of drugs targeting the same pathway in different diseases highlight the important shaping role of disease context on drug effects. Taking the soluble TACI-Fc decoy receptor atacicept as an example, this drug neutralizes both BAFF and APRIL rather than simply blocking TACI signaling. It showed some efficacy in SLE clinical trials but unexpectedly aggravated disease activity in multiple sclerosis (MS) trials, leading to premature termination (109). In the Phase III ORIGIN trial, atacicept significantly reduced proteinuria compared with placebo at week 36 (110), forming the basis for the U.S. FDA’s accelerated approval of atacicept for adult primary IgA nephropathy in July 2026^2^—the first FDA-approved BAFF/APRIL-targeting medicine. This regulatory milestone further underscores the profound context-dependent nature of BAFF/APRIL neutralization across different disease settings. This differential outcome can first be understood from the different pathological roles of B cells in the two diseases: SLE is driven primarily by B-cell hyperactivation and autoantibody production, so BAFF/APRIL neutralization by a TACI-Fc decoy receptor can directly inhibit the survival of pathogenic B cells and plasma cells; whereas MS pathogenesis is mainly driven by T-cell (especially Th1 and Th17)-mediated CNS inflammation, and the pathogenic role of B cells is more reflected in antigen presentation and cytokine secretion rather than autoantibody production. This reflects the context-dependent duality of BAFF/APRIL neutralization: in SLE, where pathogenic B cells and plasma cells drive disease, TACI-Fc decoy receptor therapy suppresses humoral autoimmunity; in MS, where T-cell-mediated CNS inflammation predominates and B cells serve primarily as antigen-presenting cells, the same intervention unexpectedly exacerbated disease activity in clinical trials (109), suggesting that the immunoregulatory roles of TACI in the central nervous system—potentially involving B-cell–T-cell crosstalk, differ fundamentally from those in peripheral autoimmune settings.

In B-cell-driven SLE, biomarker-guided precision stratification is particularly important – baseline serological activity (such as anti-dsDNA antibody positivity, low complement levels) and high disease activity (SLEDAI ≥10) have been shown to effectively predict treatment responses to biologics (including anti-BAFF agents) (111). A real-world study further showed that telitacicept treatment guided by BAFF/APRIL expression levels showed more favorable clinical responses and laboratory parameter improvements in an observational, non-head-to-head comparison involving belimumab-treated patients without expression testing, providing preliminary evidence for biomarker-based individualized treatment (95). However, the predictive value of interferon gene signature (IGS) has been inconsistent across studies, highlighting the complexity of biomarker validation (111).

In addition, B-cell depletion therapy (such as anti-CD20 antibodies) is often followed by a compensatory rise in serum BAFF levels, a phenomenon considered to be an important mechanism leading to B-cell reconstitution, autoreactive clone reactivation, and disease relapse, constituting a key challenge for monotherapy failure (4). This mechanism provides a direct theoretical basis for combining rituximab with BAFF inhibitors. Clinical observations further support this combination approach: the rebound elevation of BAFF levels after rituximab-mediated B-cell depletion is considered a major factor leading to B-cell reconstitution and disease relapse; therefore, sequential or combination therapy with rituximab and BAFF inhibitors (such as belimumab) is being explored for refractory autoimmune diseases such as pemphigus to more durably control autoreactive B cells (4, 112). How to effectively manage this BAFF rebound to avoid pathological B-cell reconstitution remains an urgent clinical issue.

To address these challenges, combination therapy strategies, particularly sequential or concomitant use of BAFF inhibitors with anti-CD20 antibodies (e.g., rituximab), have become an important clinical direction. The rationale is that combining BAFF blockade after B-cell depletion can interfere with B cell repopulation, thereby achieving more durable control of autoreactive B cells (112, 113). Preclinical and preliminary clinical evidence supports this strategy: in pemphigus (112), SLE (114, 115), and pSS (116), sequential or combination regimens have shown synergistic effects, including more profound B-cell depletion and better clinical outcomes. In addition, combining BAFF inhibitors with tacrolimus (117) or rapid glucocorticoid tapering (118) also helps achieve low disease activity and reduce glucocorticoid usage. In B-cell malignancies, combining BAFF inhibition with existing regimens (such as belimumab plus rituximab/venetoclax in CLL (1)) may also bring dual benefits by reducing tumor burden and suppressing malignant cell output (50). However, the optimal combination regimens, timing, and long-term safety still require validation in large randomized controlled trials.

Safety is another core consideration of BAFF/APRIL-targeted therapy. The approved anti-BAFF antibody belimumab has accumulated extensive safety data in adult and pediatric SLE and lupus nephritis (LN). Multiple integrated analyses have shown that compared with placebo, belimumab has similar overall adverse events, serious adverse events, and mortality rates; long-term extension studies have not identified new safety signals, both confirming its favorable benefit–risk profile (90, 119). In elderly SLE patients, its safety profile is generally consistent with the overall population (120). Pooled analyses show that common adverse events in adult patients include infections and infusion/injection reactions (90). However, post-marketing surveillance has also identified some new potential safety signals, such as dose omissions due to poor patient adherence and inappropriate dosing regimens (121). Rare but serious adverse events, such as acute pancreatitis, have been reported but are extremely rare; causality with the drug requires further confirmation (122).

For novel dual inhibitors (such as telitacicept and povetacicept), their long-term safety profiles are still being established. For example, in early studies of refractory IgA vasculitis nephritis (IgAVN) and IgA nephropathy (IgAN), telitacicept and povetacicept showed good tolerability, with serious adverse events rare but present (123, 124). Furthermore, data in special populations (such as pregnant women) remain limited; although case reports suggest that belimumab use during pregnancy may be relatively safe, the drug can cross the placenta and affect neonatal lymphocyte subsets, warranting cautious evaluation (125). In summary, as novel BAFF/APRIL-targeted therapies continue to emerge, ongoing pharmacovigilance, biomarker-based patient selection, and in-depth understanding of long-term immunological impacts are essential for maximizing therapeutic benefit and minimizing risk.

### 6.3 Emerging therapeutic technologies and iterative strategies

With the deepening understanding of the central roles of the BAFF/APRIL system in autoimmune diseases and B-cell malignancies, therapeutic strategies targeting this system are undergoing rapid iteration from single-target to multi-target synergy, and from antibody drugs to cell therapies and novel delivery systems. In the autoimmune field, more potent BAFF/APRIL dual antagonists are continuously emerging. For example, povetacicept (ALPN-303), which has been affinity-enhanced through directed evolution (126, 127), is in Phase III clinical development for IgA nephropathy (RAINIER, NCT06564142) (128).

In the treatment of B-cell malignancies, BAFF ligand- or receptor-based immunotherapies have also become important emerging strategies. Studies have shown that members of the BAFF-APRIL system (including BAFF, APRIL, and their receptors BAFF-R, BCMA, TACI) are highly expressed on various B-cell tumors and correlate with disease severity and treatment response (129). BAFF ligand-based CAR-T therapy has demonstrated potent anti-tumor potential. A BAFF ligand-based CAR-T cell capable of simultaneously targeting BAFF-R, BCMA, and TACI effectively kills multiple B-cell tumors *in vitro* and *in vivo*, including MCL, MM, and ALL, thereby minimizing the risk of treatment failure due to antigen escape (68).

Moreover, BAFF-R-targeted CAR-T cells have shown cytotoxicity in CD19-negative or resistant models such as CLL (130, 131), and because BAFF-R is not expressed on early B cell progenitors, they are expected to reduce the risk of long-term B cell aplasia (1, 132). For MM, to overcome resistance caused by BCMA antigen loss, bispecific BAFF-R/BCMA CAR-T cells have been developed. Single-cell RNA sequencing revealed that BAFF-R gene expression is upregulated in relapsed/refractory MM, and BAFF-R is expressed on earlier plasma cell differentiation stages, complementing BCMA (132). This dual-targeting CAR-T cell effectively controls the growth of heterogeneous plasma cells in preclinical models and compensates for the risk of antigen escape due to BCMA downregulation (132).

Beyond cell therapies, novel antibody drugs are also being developed. For example, an engineered BAFF-based trifunctional T-cell engager (TCE), which fuses anti-CD3 Fab with the BAFF ligand, effectively bridges T cells and BAFF receptor-expressing tumor cells, triggering robust tumor immunity in various B cell malignancy models. This TCE exhibited variable anti-tumor activity across distinct B-cell malignancy models and outperformed blinatumomab in the cited preclinical leukemia models, highlighting a tumor-type-dependent effect (133). This preclinical comparison does not establish clinical superiority. A humanized, afucosylated monoclonal antibody targeting BAFF-R, engineered to enhance ADCC, has shown potent anti-tumor activity in preclinical models of MCL and can overcome chemoresistance (49). In addition, cell membrane vesicle-based nanotechnology has been applied to target excess BAFF/APRIL in the tumor microenvironment. For example, nanovesicles displaying reconstructed BCMA can act as “decoys” to capture excess BAFF and APRIL in the bone marrow microenvironment, thereby inhibiting MM cell survival and synergizing with bortezomib (54). These emerging technologies and iterative strategies together offer a promising landscape for BAFF-targeted therapy, ranging from molecules to cells and from single agents to combinations, providing a more diversified and precise arsenal for combating autoimmune and malignant diseases.

## 7 Value of BAFF in disease diagnosis and prognosis assessment

### 7.1 As a biomarker in autoimmune diseases

The systematic analysis of BAFF’s roles in tumor immunity and autoimmune pathology described above reveals extensive associations between BAFF expression levels and disease activity, treatment response, and clinical outcomes. Based on this, BAFF shows significant serum level elevation in various autoimmune diseases, making it a highly promising biomarker for disease activity. In SLE, numerous studies have confirmed that serum BAFF levels correlate with disease activity and autoantibody titers (4). Preclinical pharmacodynamic studies demonstrate that successful BAFF suppression produces concordant reductions in circulating ligand levels, autoantibody titers, and end-organ damage indices, establishing a direct quantitative link between BAFF concentrations and reversible disease activity (86).

Furthermore, in autoimmune hepatitis (AIH) patients, pretreatment BAFF and IL-21 levels could distinguish different immunophenotypic subgroups and correlated with post-treatment responses: patients with elevated BAFF and normal IL-21 had a higher remission rate (54%), whereas those with elevated IL-21 had the lowest remission rate (0%) and were prone to progression to a PSC variant. This stratification ability suggests that BAFF combined with IL-21 may have potential value in aiding the assessment of treatment response (33). The value of BAFF as a biomarker is not only reflected in reflecting overall disease activity but also in predicting response or relapse risk after specific treatments. For example, after rituximab-mediated B-cell depletion, a sharp rise in BAFF levels (i.e., “BAFF flare”) is frequently observed, and this response is associated with increased risk of disease relapse (4). Therefore, monitoring dynamic changes in BAFF after treatment may help identify patients at high risk of relapse and inform decisions regarding subsequent BAFF-targeted therapy. In ocular cicatricial pemphigoid, serum BAFF levels are significantly elevated during active disease and decrease during remission; notably, patients who relapsed and were refractory to rituximab showed abnormally high BAFF levels, suggesting that BAFF levels may serve as an important indicator for identifying refractory cases and guiding BAFF-targeted therapy (134).

In addition to serum levels, genetic variations in the BAFF system may also serve as biomarkers for disease susceptibility or phenotype. Although in some diseases such as immunoglobulin A vasculitis (IgAV), common genetic polymorphisms of BAFF, APRIL, and their receptors do not appear to constitute major genetic risk factors (135), in other disease contexts, specific gene variants have important implications. For example, in patients with hepatitis C virus-related cryoglobulinemic vasculitis and non-Hodgkin’s lymphoma, the allele frequencies of specific single-nucleotide polymorphisms in the BAFF promoter region and BAFF-R coding region were significantly higher than in HCV-infected patients without these complications, suggesting that genetic features of BAFF/BAFF-R may constitute risk haplotypes for disease development and may help identify patient subgroups that could benefit more from anti-BAFF therapy (136). Furthermore, in Graves’ disease, estrogen may regulate BAFF expression, and BAFF levels correlate with thyroid autoantibodies and thyroid function (especially in females), providing mechanistic clues for the higher prevalence of autoimmune thyroid disease in women and suggesting that the interaction between BAFF and sex hormones may differ between male and female patients (137).

In summary, BAFF and its pathway-related molecules, through detection of circulating levels, cellular expression, or genetic features, show multifaceted biomarker potential in diagnostic stratification, activity assessment, treatment response prediction, and relapse risk monitoring in autoimmune diseases.

### 7.2 Significance in tumor prognosis and treatment response prediction

BAFF expression levels in various tumors are closely associated with patient prognosis and treatment response, making it a potential predictive biomarker. In hematological malignancies, the soluble form of BCMA, sBCMA – generated by γ-secretase cleavage of BCMA (27) – is emerging as a promising biomarker. In MM, serial sBCMA measurements provide a quantitative readout of tumor burden and treatment response, with elevated baseline levels predicting inferior outcomes (28, 138). Its accumulation in serum also poses a pharmacokinetic challenge for BCMA-directed immunotherapies by limiting ligand accessibility to cellular target (138).

In hematological malignancies, elevated BAFF levels are often associated with poor prognosis and disease progression. For example, in relapsed/refractory MM patients, serum BAFF levels were significantly higher than in newly diagnosed patients, and levels decreased significantly in responders after induction therapy but showed no significant change in non-responders, suggesting that BAFF is not only associated with disease stage but may also serve as a potential biomarker for monitoring treatment response (139). In primary CNS B-cell lymphoma, changes in cerebrospinal fluid BAFF levels correlated with treatment response: levels decreased in responders and might increase in non-responders, suggesting utility for monitoring efficacy (140). In addition, in AIDS-related non-Hodgkin lymphoma, elevated pretreatment plasma BAFF levels were associated with shorter overall survival and progression-free survival in univariate analysis, suggesting its potential as a prognostic biomarker (141).

In the solid tumor field, BAFF also shows important prognostic significance. In colorectal cancer tissues, BAFF expression is significantly elevated and, together with APRIL and MMP-3, constitutes a pathway associated with unfavorable clinicopathological features (142). For patients receiving immune checkpoint inhibitors, BAFF levels may affect toxicity. In non-small cell lung cancer (NSCLC) patients, elevated pretreatment serum BAFF levels were a strong risk factor for immune-related adverse events during atezolizumab treatment (143). In metastatic melanoma patients, higher plasma BAFF levels correlated with lower response rates to anti-PD-1 immunotherapy, suggesting that BAFF may influence treatment response by modulating immune cells (such as monocytes) in the tumor microenvironment (58). Moreover, in multiple cancer types including melanoma, NSCLC, and breast cancer, the presence of TLS is associated with prolonged patient survival and improved response to immunotherapy. BAFF has been shown to be required for TLO formation in experimental kidney-transplant models (39). Whether BAFF similarly supports tumor-associated TLS remains an inference that requires direct validation in human tumors. These findings collectively indicate that BAFF is not only a marker of tumor aggressiveness but may also become a key molecule for predicting whether patients will benefit from specific therapies (especially immunotherapies).

Beyond its role as a prognostic marker, BAFF and its signaling pathway are also involved in therapeutic resistance of tumor cells, further highlighting its dual potential as both a therapeutic target and a response predictor. In MCL, elevated serum BAFF at diagnosis independently predicts inferior chemotherapy response and earlier relapse, while BAFF-R-directed antibody co-administration restores chemosensitivity in refractory cases (49). Similarly, in MM, BAFF-R expression increases in relapsed/refractory cases and can serve as a prognostic marker for predicting long-term complete remission, making it a complementary CAR-T target to BCMA to prevent antigen escape (132). In hairy cell leukemia variant, BAFF induces chemoresistance to purine nucleoside analogs (such as cladribine) by activating the non-canonical NF-κB pathway and upregulating pro-survival genes (144). In triple-negative breast cancer, high intratumoral BAFF expression is associated with an immunosuppressive signature and inferior anti-PD-1 response, supporting its evaluation as a stratification biomarker for immunotherapy selection (55). In summary, monitoring BAFF levels and pathway activity may help identify high-risk patients likely to be resistant to standard therapies and provide a rationale for combination strategies targeting the BAFF/APRIL axis to improve clinical outcomes.

### 7.3 From biomarker to therapeutic decision: translational status and gaps

The above evidence indicates that BAFF system molecules already possess value for disease stratification and prognosis prediction, but their translation from “diagnostic aid” to “therapeutic decision-making tool” is still in its early stages, facing multiple bottlenecks including analytical standardization, prospective validation, and regulatory qualification. In SLE, this translation has achieved preliminary breakthroughs. SLE patients treated with telitacicept guided by dual BAFF/APRIL positivity, despite having higher baseline disease activity, achieved higher complete remission rates, more significant improvements in complement C3 and anti-dsDNA antibody reductions, and greater glucocorticoid sparing (95). In addition, in SLE, baseline serum IgA2 anti-dsDNA antibody levels were identified as a marker of response to belimumab after rituximab therapy, and their decline paralleled the reduction of IgA2 expression in IgA2 plasmablasts, further validating their value as theranostic biomarkers (145).

However, most of these studies derive from retrospective analyses or real-world cohorts and lack rigorous validation in prospective randomized controlled trials (RCTs) as companion diagnostics. In the field of kidney transplantation, SNPs in BAFF system genes have been explored as genetic markers for predicting clinical outcomes. It was found that the AA genotype of the TNFSF13 rs3803800 polymorphism was significantly associated with increased risk of acute rejection and shorter rejection-free survival in kidney transplant recipients, suggesting its potential as a biomarker for predicting acute rejection (146). In addition, for highly sensitized kidney transplant candidates, belimumab can mobilize memory B cells into the circulation, thereby more comprehensively revealing HLA-specific memory B cell profiles; this may improve pre-transplant immunological risk stratification and facilitate safer identification of removable HLA-specific contraindications, increasing transplantation opportunities (147).

However, not all disease areas have equal conditions for biomarker translation. In the renal field, the treatment of IgAN is undergoing a paradigm shift from supportive care to targeted therapy, with BAFF/APRIL pathway inhibitors (such as sibeprenlimab, telitacicept, etc.) being important emerging therapies (148). The key to achieving precision medicine lies in developing biomarkers that reflect disease activity and guide individualized therapy. Currently, the IgAN international risk prediction tool is the gold standard for predicting 5-year renal outcomes, but biomarkers that accurately reflect disease activity remain to be validated, which is critical for optimizing individualized treatment strategies (149). How to integrate BAFF system markers into clinical decision pathways for such diseases, achieving a closed loop from “stratification diagnosis” to “stratified intervention,” remains a key bottleneck in translational medicine.

In summary, the translation of BAFF system markers into clinical practice faces multiple hurdles. These include the transition from association discovery to mechanistic validation, from retrospective single-center studies to prospective multi-center cohorts, and from total protein quantification to oligomer-specific detection. Establishing standardized assays, validating findings in rigorously designed prospective trials, and incorporating regulatory science frameworks are essential steps. Only through such efforts can BAFF evolve from a research parameter into a precision medicine tool that guides clinical decisions.

## 8 Conclusions

Limitations of this review. Several limitations qualify the conclusions above. First, this is a narrative, hypothesis-driven review rather than a systematic review. Search terms and study selection were directed by the four-variable framework, which may have introduced selection bias toward findings consistent with the proposed model. Second, the evidence base is heterogeneous and spans transgenic mouse models, *in vitro* B-cell assays, observational human cohorts, and randomized trials of different designs. Cross-tier comparison therefore remains inferential. Third, direct measurement of BAFF oligomeric state in diseased human tissues remains technically limited, and most disease-specific oligomeric assignments are inferred rather than directly quantified *in vivo*. Fourth, serum BAFF is not interchangeable with local tissue concentration. The concentration axis extrapolates from serum measurements to local exposure without direct spatial validation.

BAFF’s functional duality across cancer and autoimmunity is proposed to be jointly shaped by four variables, namely ligand concentration, oligomeric form, cellular source, and receptor availability (**Table 1**). This framework integrates correlative and preclinical evidence but has not been validated as a causal law in any single experimental system. By structuring the evidence around these four mechanistic variables—rather than around disease categories—this review bridges the cancer–autoimmunity divide that has historically siloed BAFF research. In autoimmune diseases, excess BAFF breaks B-cell tolerance and drives pathogenic autoantibody production; in hematological tumors, the BAFF/BAFF-R axis directly supports malignant clone survival and chemoresistance; whereas in solid tumors, its net effect depends on the integration of local concentration and immune networks. The “inverted U-shaped” concentration-effect relationship is presented as a conceptual working hypothesis without defined concentration thresholds for reconciling contradictory findings in solid tumors; its validation requires direct *in vivo* titration studies, oligomer-specific assays, and spatial single-cell omics. None of the cited studies have performed complete low-to-intermediate-to-high BAFF titrations in the same experimental system with other variables held constant. Autoimmune disease configurations provide conceptual support for this framework, which was initially developed to reconcile contradictory observations in tumors.

Disease-specific oligomeric assignments remain inferred and require direct *in vivo* quantification and oligomer-specific assays for validation.

At the clinical translation level, targeting the BAFF/APRIL axis has achieved breakthrough progress, as exemplified by the success of belimumab in systemic lupus erythematosus and the positive Phase 3 results of telitacicept in IgA nephropathy (96). However, treatment response heterogeneity, compensatory BAFF elevation following B-cell depletion, and the long-term impact of immunosuppression on infection susceptibility and vaccine responses remain core bottlenecks. Combination strategies—particularly sequential or concomitant regimens of BAFF inhibitors with anti-CD20 antibodies—and biomarker-guided patient stratification represent the most promising directions to overcome these challenges.

A key future priority is the development of oligomer-specific BAFF assays, which will be essential for translating this framework into precision theranostics.

## Footnotes

1 Telitacicept was approved by the China National Medical Products Administration (NMPA) for SLE (March 2021), rheumatoid arthritis (July 2024), generalized myasthenia gravis (May 2025), conditionally for IgA nephropathy, and fully approved for Sjögren's disease (June 2026). Available at: https://www1.hkexnews.hk/listedco/listconews/sehk/2022/0919/2022091900524.pdf; https://www1.hkexnews.hk/listedco/listconews/sehk/2025/0327/2025032701703.pdf; https://www.hkexnews.hk/listedco/listconews/sehk/2025/0527/2025052700303.pdf; https://www1.hkexnews.hk/listedco/listconews/sehk/2026/0608/2026060801564.pdf; https://www1.hkexnews.hk/listedco/listconews/sehk/2026/0608/2026060801486.pdf.

2 U.S. Food and Drug Administration. FDA approves new treatment to reduce proteinuria in adults with primary immunoglobulin A nephropathy (atacicept, Trutakna). (2026). Available at: https://www.fda.gov/drugs/news-events-human-drugs/fda-approves-new-treatment-reduce-proteinuria-adults-primary-immunoglobulin-nephropathy.
